# Supplementary material for: Predicting visual function by interpreting a neuronal wiring diagram
Source: Nature. 2024 Oct 2;634(8032):113–23. doi: 10.1038/s41586-024-07953-5 (PMC11446822; doi:10.1038/s41586-024-07953-5)
Supplement: Supplementary file 6 — CRF and ERF predictions for individual TmY4 and TmY9 cells. Analogous to Supplementary Data 3, but for TmY target types. Shown are the top four monosynaptic pathways, the strongest pathway passing through each of the top ten intermediary types (ranking from Extended Data Fig. 7), and the trisynaptic pathway Tm1–TmY–Dm3–TmY (see the section entitled Prediction of spatial normalization). [file 41586_2024_7953_MOESM6_ESM.zip › DataS4/TmY9qΓèÑ/720575940628123880.pdf]

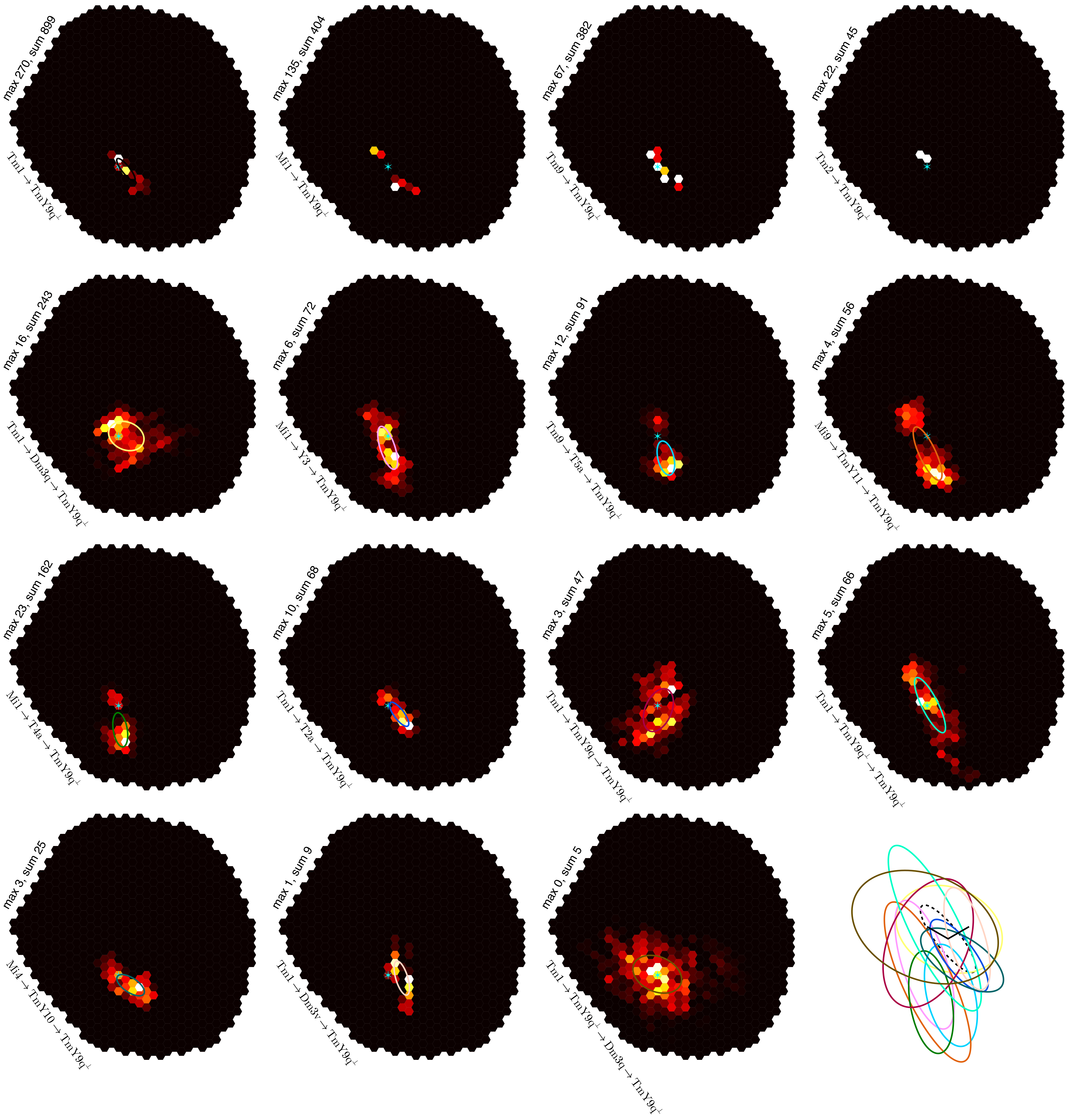

max 270, sum 899
Tm1 → TmY9q⊥
max 135, sum 404
Mi1 → TmY9q⊥
max 67, sum 382
Tm9 → TmY9q⊥
max 22, sum 45
Tm2 → TmY9q⊥
max 16, sum 243
Tm1 → Dm3q → TmY9q⊥
max 6, sum 72
Mi1 → Y3 → TmY9q⊥
max 12, sum 91
Tm9 → T5a → TmY9q⊥
max 4, sum 56
Mi9 → TmY11 → TmY9q⊥
max 23, sum 162
Mi1 → T4a → TmY9q⊥
max 10, sum 68
Tm1 → T2a → TmY9q⊥
max 3, sum 47
Tm1 → TmY9q → TmY9q⊥
max 5, sum 66
Tm1 → TmY9q⊥ → TmY9q⊥
max 3, sum 25
Mi4 → TmY10 → TmY9q⊥
max 1, sum 9
Tm1 → Dm3v → TmY9q⊥
max 0, sum 5
Tm1 → TmY9q⊥ → Dm3q → TmY9q⊥
